# Immunohistochemical analysis of 147 cases of low-grade endometrial stromal sarcoma: refining the immunohistochemical profile of LG-ESS on a large, molecularly confirmed series

**DOI:** 10.1007/s00428-025-04026-4

**Published:** 2025-01-21

**Authors:** Miroslava Flídrová, Pavel Dundr, Romana Vránková, Kristýna Němejcová, David Cibula, Renata Poncová, Květoslava Michalová, Jiří Bouda, Jan Laco, Munachiso Ndukwe, Janusz Ryś, Mariusz Książek, Alberto Berjon, Ignacio Zapardiel, Ivan Franin, Antonela Njavro, Jitka Hausnerová, Petra Bretová, Vladimír Židlík, Jaroslav Klát, Zoard Tibor Krasznai, Robert Poka, Nataliya Volodko, Iryna Yezhova, Radovan Pilka, Radim Marek, Georgina Kolnikova, Milan Krkoška, Michael Halaška, Jana Drozenová, Dagmar Dolinská, Vladimír Kalist, Marcin Bobiński, Marta Ostrowska-Leśko, Magdalena Bizoń, Włodzimierz Sawicki, Maciej Stukan, Karolina Grabowska, Marcin Jędryka, Tymoteusz Poprawski, Simona Stolnicu, Mihai Emil Căpîlna, Zuzana Špůrková, Michal Zikán, Francesca Ciccarone, Giovanni Scambia, Archil Sharashenidze, Miranda Gudadze, Tetiana Piatnytska, Ihor Varchak, Michaela Kendall Bártů

**Affiliations:** 1 Department of Pathology, First Faculty of Medicine, Charles University and General University Hospital in Prague, Prague, Czech Republic; 2 Department of Gynaecology, Obstetrics and Neonatology, First Faculty of Medicine, Charles University and General University Hospital in Praque, Prague, Czech Republic; 3 Department of Pathology, Faculty of Medicine in Pilsen, Charles University, Pilsen, Czech Republic; 4 Biopticka laboratory, University Hospital in Pilsen, Czech Republic, Pilsen, Czech Republic; 5 Department of Gynecology and Obstetrics, Faculty of Medicine in Pilsen, Charles University, Pilsen, Czech Republic; 6 University Hospital in Pilsen, Pilsen, Czech Republic; 7 The Fingerland Department of Pathology, Charles University Faculty of Medicine in Hradec Kralove and University Hospital Hradec Kralove, Hradec Kralove, Czech Republic; 8 Department of Obstetrics and Gynecology, Department of Oncology and Radiotherapy, Charles University Faculty of Medicine in Hradec Kralove and University Hospital Hradec Kralove, Hradec Kralove, Czech Republic; 9 Department of Tumor Pathology, Maria Sklodowska-Curie National Reaserch Institute of Oncology, Cracow Branch, Cracow, Poland; 10 Department of Pathology, University Hospital Crosshouse, Kilmarnock, Scotland; 11 Pathology Department, La Paz University Hospital, Madrid, Spain; 12 Gynecologic Oncology Unit, La Paz University Hospital, Madrid, Spain; 13 Ljudevit Jurak Department of Pathology and Cytology, Sestre milosrdnice University Hospital Centre, Zagreb, Croatia; 14 Department of Oncology and Nuclear Medicine, Sestre milosrdnice University Hospital Centre, Zagreb, Croatia; 15 Department of Pathology, University Hospital Brno and Medical Faculty, Masaryk University, Brno, Czech Republic; 16 Department of Gynecology and Obstetrics, University Hospital Brno and Faculty of Medicine, Masaryk University, Brno, Czech Republic; 17 Department of Clinical and Molecular Pathology and Medical Genetics, University Hospital and Faculty of Medicine, University of Ostrava, Ostrava, Czech Republic; 18 Department of Obstetric and Gynecology, University Hospital and Faculty of Medicine, University of Ostrava, Ostrava, Czech Republic; 19 Department of Gynecology and Obstetrics, Faculty of Medicine, University of Debrecen, Debrecen, Hungary; 20 Lviv Regional Oncological Center, Department of Oncology and Radiology Danylo Halytsky, Lviv national medical university, Lviv, Ukraine; 21 Department of Obstetrics and Gynecology, Faculty of Medicine and Dentistry, Palacky University, University Hospital Olomouc, Olomouc, Czech Republic; 22 Faculty of Humanities, Tomas Bata University in Zlín, Zlín, Czech Republic; 23 Department of Pathology, National Oncology Institute, Bratislava, Slovakia; 24 Department of Gynecologic Oncology, National Oncology Institute, Bratislava, Slovakia; 25 Department of Obstetrics and Gynecology, 3rd Medical Faculty, Charles University in Prague and University Hospital Kralovske Vinohrady, Prague, Czech Republic; 26 Department of Pathology, 3rd Medical Faculty, Charles University in Prague and University Hospital Kralovske Vinohrady, Prague, Czech Republic; 27 Pathological and Anatomical Department, Tomas Bata Regional Hospital, Zlín, Czech Republic; 28 Department of Gynecology and Obstetrics, Tomas Bata Regional Hospital, Zlín, Czech Republic; 29 Chair and Department of Oncologic Gynecology and Gynecology, Medical University of Lublin, Lublin, Poland; 30 Chair and Department of Toxicology, Medical University of Lublin, Lublin, Poland; 31 LUX MED Oncology Hospital, Warsaw, Poland; 32 Chair and Department of Obstetrics, Gynaecology and Gynaecological Oncology, Medical Univerity of Warsaw, Warsaw, Poland; 33 Department of Gynecological Oncology, Pomeranian Hospitals, Gdynia, Poland; 34 Surgical Oncology Clinic, Medical University in Gdansk, Gdansk, Poland; 35 Department of Gynecological Oncology, Wroclaw Medical University, Wroclaw, Poland; 36 Department of Oncological Gynecology, Lower Silesian Oncology, Pulmonology and Hematology Center, Wroclaw, Poland; 37 Department of Pathology, University of Medicine, Pharmacy, Sciences and Technology GE Palade, Targu Mures, Romania; 38 Department of Gynecology, University of Medicine, Pharmacy, Sciences and Technology GE Palade, Targu Mures, Romania; 39 Department of Pathology, University Hospital Bulovka, Prague, Czech Republic; 40 Department of Gynecology and Obstetrics, Charles University - First Faculty of Medicine and University Hospital Bulovka, Prague, Czech Republic; 41 Gynecologic Oncology Unit, Department of Woman and Child Health and Public Health, Fondazione Policlinico Universitario ’A. Gemelli’ IRCCS, Rome, Italy; 42 Section of Obstetrics and Gynecology, University Department of Life Sciences and Public Health, Università Cattolica del Sacro Cuore, Rome, Italy; 43 Caucasus Medical Centre, Onco-gynecological Department, Tbilisi, Georgia; 44 Caucasus Medical Centre, Megalab, Tbilisi, Georgia; 45 Department of Gynecologic Oncology, Khmelnytskyi regional anti tumor center, Khmelnytskyi, Ukraine

**Keywords:** Low-grade endometrial stromal sarcoma, LG-ESS, Immunohistochemistry, Endometrial stromal markers, Smoothelin

## Abstract

Low-grade endometrial stromal sarcoma (LG-ESS) can present diagnostic challenges, due to its overlapping morphological features with other uterine mesenchymal tumors. Misdiagnosis rates remain significant, and immunohistochemical data for LG-ESS are limited to small series and inconsistent antibody panels. This study aimed to refine the IHC profile of LG-ESS by analyzing a large, molecularly confirmed series of 147 cases using a panel of 24 antibodies, including newer markers like transgelin and smoothelin. CD10 and IFITM1, key endometrial stromal markers, were expressed in 86% (92% of those extensively) and 69% (60% of those extensively) of cases, with fusion-positive tumors showing significantly higher expression. Smooth muscle markers (α-SMA, desmin, h-caldesmon, calponin, transgelin) were variably expressed, predominantly in focal or low-intensity patterns, with α-SMA reaching the highest frequency of expression (44%). However, the intensity of smooth muscle marker expression was usually very low. Smoothelin was rarely expressed. Hormone receptors were frequently positive, with PR showing a higher frequency (92% vs. 83%) and intensity than ER. Markers like S-100, HMB45, and CD117 were largely negative; all tumors were p53 wild-type, with preserved SMARCB1/SMARCA4 expression and ALK and ROS1 negativity. This work represents the largest molecularly validated IHC study on LG-ESS, providing a robust diagnostic profile for routine pathology. By addressing key diagnostic limitations and examining newer markers, our study supports a more standardized approach to diagnosing LG-ESS and underscores the value of immunohistochemical panels, particularly in fusion-negative tumors where diagnosis relies on morphological and immunohistochemical interpretation. These findings contribute critical data for improving diagnostic accuracy.

**Supplementary Information:**

The online version contains supplementary material available at 10.1007/s00428-025-04026-4.

## Introduction

Low grade endometrial stromal sarcoma (LG-ESS) is a rare malignant mesenchymal tumor which morphologically resembles proliferative phase endometrial stroma and exhibits an infiltrative growth pattern [1]. Endometrial stromal sarcomas constitute up to 1% of all uterine cancers and 6–20% of all uterine sarcomas, with LG-ESS representing the second most common uterine sarcoma after leiomyosarcoma [2, 3]. While most cases affect the uterine body, primary extrauterine tumors can also occur, often associated with endometriosis, making diagnosis more challenging [4, 5].

In most cases, LG-ESS can be reliably diagnosed based solely on morphology due to its distinct appearance. However, in some cases, LG-ESS can exhibit significant morphological heterogeneity with a wide range of histological patterns, which can also be reflected in their immunohistochemical profile [6]. Some less common, variant morphologies include smooth muscle-like differentiation, fibroblastic and/or myxoid features, sex cord-like structures, pseudopapillary formations, clear cell change, skeletal muscle differentiation, adipocytic metaplasia, angiomatous pattern, and cells with rhabdoid appearance or bizarre nuclei [7–11].

Therefore, the differential diagnosis of LG-ESS primarily includes cellular leiomyoma (and potentially other smooth muscle tumors), inflammatory myofibroblastic tumor (IMT), and uterine tumors resembling sex cord-stromal tumors (UTROSCT) [6, 8, 9, 12–15]. Some cases with smooth muscle differentiation exhibit a characteristic “starburst” pattern, with collagen fibers radiating throughout the tumor, while others may show dispersed collagen plaques or myxoid changes, which can mimic the regressive changes found in leiomyoma or a myxoid leiomyosarcoma [7]. The presence of epithelioid/round cell differentiation may raise suspicion for high grade endometrial stromal sarcoma (HG-ESS) or perivascular epithelioid cell tumor (PEComa). In some cases, solitary fibrous tumor (SFT), gastrointestinal stromal tumor (GIST), or adenosarcoma can also stand in the differential diagnosis. Moreover, recently described sarcomas with the *KAT6B/A::KANSL1* fusion usually have overlapping features between LG-ESS/endometrial stromal nodule (ESN) and smooth muscle tumors [16–19].

With the increasing accessibility of molecular genetic methods, especially next generation sequencing (NGS), and the expanding knowledge of recurrent genetic alterations in uterine mesenchymal neoplasms, identifying characteristic fusions has greatly enhanced diagnostic accuracy. Up to 75% of LG-ESS harbor recurrent fusions, with *JAZF1::SUZ12* being the most common, followed by *JAZF1::PHF1, EPC1::PHF1*, and *MEAF6::PHF1* [6, 8, 20]. However, that leaves almost a quarter of LG-ESS which are not currently associated with any known recurrent genetic alteration. In a routine diagnostic setting, access to NGS is still rather limited and often varies between countries, with the added question of financial availability. As such, immunohistochemistry as a method which is vastly more accessible still remains a vital tool in reaching the correct diagnosis.

We conducted an extensive immunohistochemical analysis on a large, molecularly examined cohort of 147 LG-ESS, all subjected to rigorous central review. Our study tested a wide panel of immunohistochemical markers including sex cord-stromal markers, smooth muscle markers, hormone receptors, and selected novel antibodies, some of which have not been assessed in LG-ESS cohorts of this size. Our goal was to define the immunohistochemical profile of the largest cohort of LG-ESS to date, thus providing a practical diagnostic guide for pathologists, especially in settings where molecular testing is unavailable.

## Materials and methods

### Samples

The samples represent a part of the cohort assembled under the Rare Gynecological Sarcoma (REGYS) study, which is an international project involving 23 participating institutions from 10 countries, consisting mostly of members of the Central and Eastern Gynecology Oncology Group (CEEGOG). The project included a detailed assessment of the morphological features, immunohistochemical analysis, and DNA and RNA NGS analysis. A detailed description of the project, the overall cohort assembled, and the molecular results are provided in a different study (currently under review) and are not described here. Briefly, a central review was performed on all hematoxylin and eosin (HE)-stained slides available for individual cases by two experienced gynecopathologist specialists (PD, MKB). The required number of representative slides from the co-operating institutions for each case was 2 (*n* = 100). In those cases which came from the archives of our department (as well as cases which were sent to us as consultations, *n* = 47), the evaluation was performed on all slides available from the entire biopsy/resected specimen. The morphological evaluation was combined with the results of the immunohistochemical analysis and molecular testing to reach the correct diagnosis.

An immunohistochemical (IHC) examination with a broad panel of 24 selected antibodies was performed for each tumor. Only cases unequivocally diagnosed as LG-ESS after the central review were included in the study, resulting in a final sample set of 147 cases. Of these 147 cases, 133 had RNA NGS results available which showed that 101 cases (75.9%) harbored a recurrent fusion. The most common alteration was the *JAZF1::SUZ12* fusion found in 67 cases (66.3% of all fusions), followed mainly by *JAZF1::PHF1* (*n* = 9), *MEAF6::PHF1* (*n* = 8), and *EPC1::PHF1* (*n* = 4).

The study was performed on formalin-fixed, paraffin embedded (FFPE) tissue blocks. Hematoxylin and eosin-stained slides from the FFPE tissue blocks were reviewed, and tissue microarrays (TMAs) were constructed from suitable tumor areas. Two tissue cores of a 2.00 mm diameter were extracted from each FFPE tissue block using the TMA instrument TMA Master (3DHISTECH Ltd., Budapest, Hungary).

### Immunohistochemical analysis

Immunohistochemistry was performed on 4 µm thick section using the TMAs. In cases where the TMA approach was not possible, particularly due to small tumor size or technical difficulties during sample processing, whole-tissue sections were used where possible. The IHC was evaluated independently by two pathologists (MKB, MF). The antibodies used for IHC examination were selected based on their diagnostic utility, with an emphasis on ruling out the other entities most commonly involved in the differential diagnosis. The whole panel was comprised of estrogen receptor (ER), progesterone receptor (PR), alfa-smooth muscle actin (α-SMA), desmin, h-caldesmon, calponin, CD10, IFITM1, transgelin, BCOR, BCORL1, NTRK, S-100, HMB45, CD117, WT1, SMARCA4 (BRG1), SMARCB1 (INI1), SMARCA2, ALK, ROS1, p53, smoothelin, and cyclin D1. The complete list of antibodies used, including their clones, dilution, and manufacturers is provided in Table 1.

**Table 1:** List of antibodies used for immunohistochemical analysis

Antibody	Clone	Dilution	Producer	Platform	Detection	Evaluated expression
ALK	D5F3	1:100	Cell Signaling Technology, Danvers, Massachusetts, USA	Ventana BenchMark ULTRA (Roche, Basel, Switzerland)	OptiView	Cytoplasmic
BCOR	C-10	1:50	Santa Cruz Biotechnology, Dallas, Texas, USA	Ventana BenchMark ULTRA (Roche, Basel, Switzerland)	OptiView	Nuclear
BCORL 1	Polyclonal rabbit	1:200	Atlas antibodies, Bromma, Sweden	Dako Omnis, (Agilent, Santa Clara, CA, USA)	EnVision FLEX (Dako) + Linker	Nuclear
SMARCA4 (BRG1)	EPNCIR 111A	1:200	Abcam, Cambridge, UK	Dako Omnis, (Agilent, Santa Clara, CA, USA)	EnVision FLEX (Dako)	Nuclear
H-caldesmon	h-CALD	1:800	Santa Cruz Biotechnology, Dallas, Texas, USA	Dako Omnis, (Agilent, Santa Clara, CA, USA)	EnVision FLEX (Dako)	Cytoplasmic
Calponin	CALP	1:400	Dako, Glostrup, Denmark	Dako Omnis, (Agilent, Santa Clara, CA, USA)	EnVision FLEX (Dako)	Cytoplasmic
CD10	56C6	1:50	Novocastra, Leica Biosystems, Wetzlar, Germany	Ventana BenchMark ULTRA (Roche, Basel, Switzerland)	OptiView	Cytoplasmic
CD117	c-kit	1:200	Dako, Glostrup, Denmark	Ventana BenchMark ULTRA (Roche, Basel, Switzerland)	UltraView	Cytoplasmic
Cyclin D1	EP 12	RTU	Dako, Glostrup, Denmark	Dako Omnis, (Agilent, Santa Clara, CA, USA)	EnVision FLEX (Dako)	Nuclear, cytoplasmic
Desmin	D33	1:200	Dako, Glostrup, Denmark	Ventana BenchMark ULTRA (Roche, Basel, Switzerland)	OptiView	Cytoplasmic
ER	SP1	1:200	Zytomed Systems GmbH, Berlin, Germany	Ventana BenchMark ULTRA (Roche, Basel, Switzerland)	OptiView	Nuclear
HMB45	HMB 45	1:50	Dako, Glostrup, Denmark	Dako Omnis, (Agilent, Santa Clara, CA, USA)	EnVision FLEX (Dako)	Cytoplasmic
IFITM1	Polyclonal rabbit	1:400	Abcam, Cambridge, UK	Dako Omnis, (Agilent, Santa Clara, CA, USA)	EnVision FLEX (Agilent)	Cytoplasmic
SMARCB1 (INI1)	MRQ-27	RTU	Cell Marque, Rocklin, CA, USA	Ventana BenchMark ULTRA (Roche, Basel, Switzerland)	OptiView	Nuclear
p53	DO-7	1:400	Dako, Glostrup, Denmark	Ventana BenchMark ULTRA (Roche, Basel, Switzerland)	OptiView	Nuclear, cytoplasmic
PR	clone 16	1:100	Novocastra, Leica Biosystems, Wetzlar, Germany	Ventana BenchMark ULTRA (Roche, Basel, Switzerland)	OptiView	Nuclear
ROS1	D4D6	1:100	Cell Signaling Technology, Danvers, Massachusetts, USA	Ventana BenchMark ULTRA (Roche, Basel, Switzerland)	OptiView	Cytoplasmic
S-100	4C4.9	1:400	DCS, Hamburg, Germany	Dako Omnis, (Agilent, Santa Clara, CA, USA)	EnVision FLEX (Agilent)	Cytoplasmic
α- SMA	1A4	1:1600	Dako, Glostrup, Denmark	Dako Omnis, (Agilent, Santa Clara, CA, USA)	EnVision FLEX (Agilent)	Cytoplasmic
SMARCA2		1:800	Atlas antibodies, Bromma, Sweden	Dako Omnis, (Agilent, Santa Clara, CA, USA)	EnVision FLEX (Dako) + Linker	Nuclear
Smoothelin	R4A	1:50	Zeta Corporation, Monrovia, CA, USA	Ventana BenchMark ULTRA (Roche, Basel, Switzerland)	OptiView	Cytoplasmic, nuclear
NTRK	EPR17341	1:100	Abcam, Cambridge, UK	Ventana BenchMark ULTRA (Roche, Basel, Switzerland)	OptiView	Nuclear, cytoplasmic
Transgelin	2A10C2	1:300	Cell Marque, Rocklin, CA, USA	Ventana BenchMark ULTRA (Roche, Basel, Switzerland)	OptiView	Cytoplasmic
WT1	6F-H2	1:400	BioSB, Santa Barbara, CA, USA	Dako Omnis, (Agilent, Santa Clara, CA, USA	EnVision FLEX (Agilent)	Nuclear, cytoplasmic

*RTU* ready to use

The immunohistochemical results were evaluated based on the overall percentage of positivity (0–100%). Cases were classified as negative (complete absence of staining or < 1% of positive tumor cells), 1 + (1–25% of positive tumor cells), 2 + (26–50% of positive tumor cells), or 3 + (> 50% of positive tumor cells). For the antibodies NTRK and smoothelin, the staining was evaluated independently as both nuclear and cytoplasmic, for WT1 only the nuclear staining was evaluated. The immunohistochemical results were also assessed using the H-score method previously described by others [21]. This method incorporates both the percentage of positive cells and the staining intensity (1 + for weak intensity, 2 + for moderate, and 3 + for strong). The final H-score is calculated by adding the multiplication of the different staining intensities according to the following formula: [1 x (% of cells 1 +)] + [2x (% of cells 2 +)] + [3x (% of cells 3 +)], resulting in an H-score value of 0–300. To characterize expression in terms of positive and negative cases, a cut-off value of 1% was used (positive: ≥ 1% of cells showing expression). For comparing the IHC expression within the LG-ESS cohort based on the presence of fusion, the cut-off value was modified to 5% to account for minor non-specific staining variations.

### Statistical analyses

All statistical analyses were conducted using R software, version 4.3.3 (2024–02–29). Standard descriptive statistics were applied to summarize the dataset: Categorical variables were reported as frequencies and percentages, and continuous variables were described using means with standard deviation (SD) or medians with interquartile range. Correlations between the expression of IHC markers (categorized as positive vs. negative) and fusion status (presence vs. absence of fusion) were assessed using Pearson’s chi-squared test or Fisher´s Exact test based on expected values. All tests were two-sided, and a *p* value < 0.05 was considered statistically significant.

## Results

**Fig. 1:**
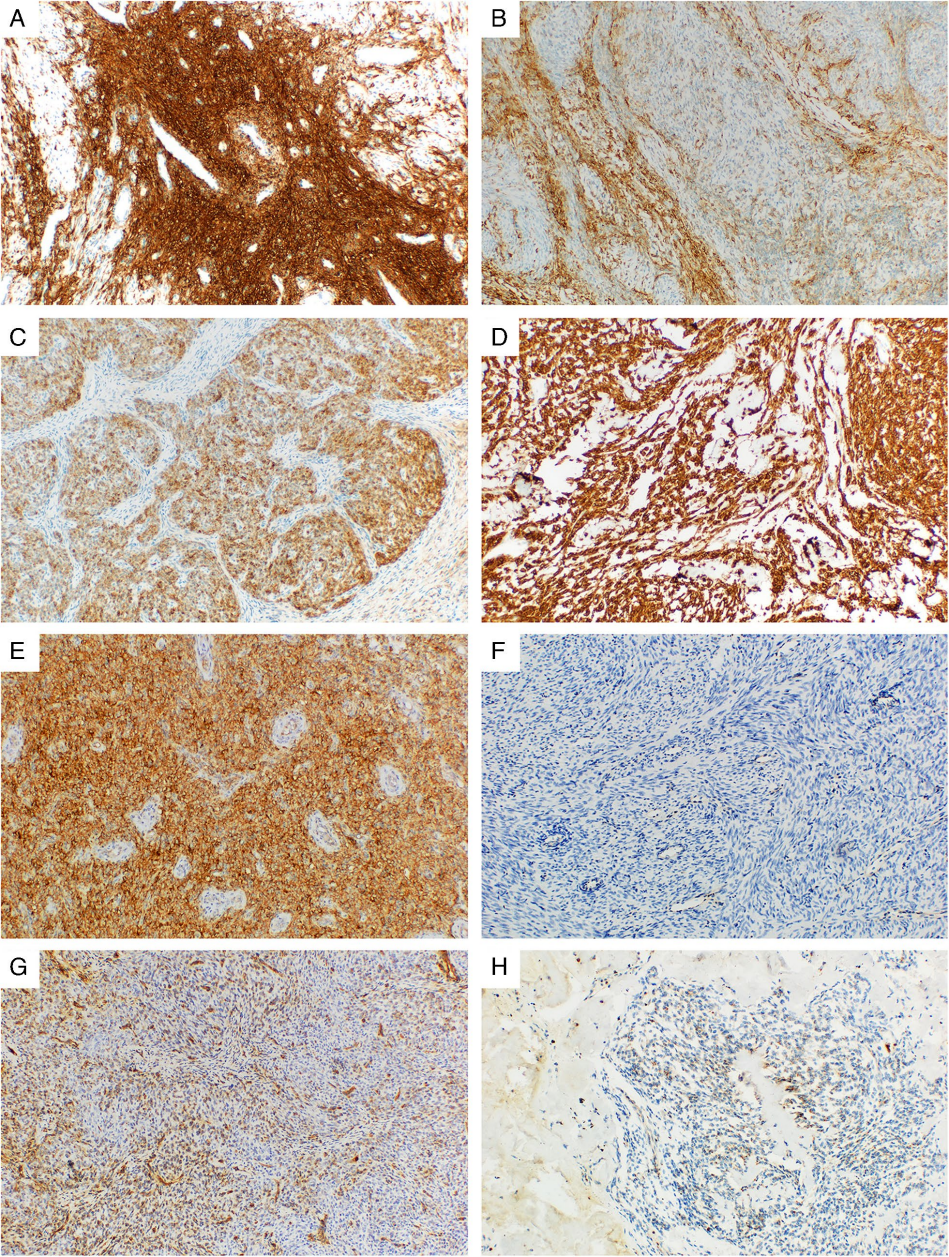
Examples of CD10 and IFITM1 expression in different morphological variants of LG-ESS. All microphotographs taken at 100 × magnification. **A** Diffuse, strong expression of CD10 in a usual LG-ESS (no fusion detected). **B** Focal expression of CD10 of a variable intensity in a case with predominant smooth-muscle morphology (*JAZF1::SUZ12* fusion). **C** Moderate to strong CD10 expression in an LG-ESS with a sex cord stromal-like morphology (*JAZF1::SUZ12* fusion). **D** Diffuse, strong expression of CD10 in a myxoid LG-ESS (*JAZF1::SUZ12* fusion). **E** Diffuse and strong expression of IFITM1 in LG-ESS with a usual morphology (*SVIL::EPC1* fusion). **F** Complete IFITM1 negativity in a case with predominantly smooth muscle morphology (*JAZF1::SUZ12* fusion). **G** Disperse IFITM1 expression of a variable intensity in an LG-ESS with sex cord stromal-like morphology. Same case as depicted in 1C (*JAZF1::SUZ12* fusion). **H** Focal, occasional granular expression of IFITM1 in a case with myxoid features. Same case as depicted in 1D (*JAZF1::SUZ12* fusion)

**Fig. 2:**
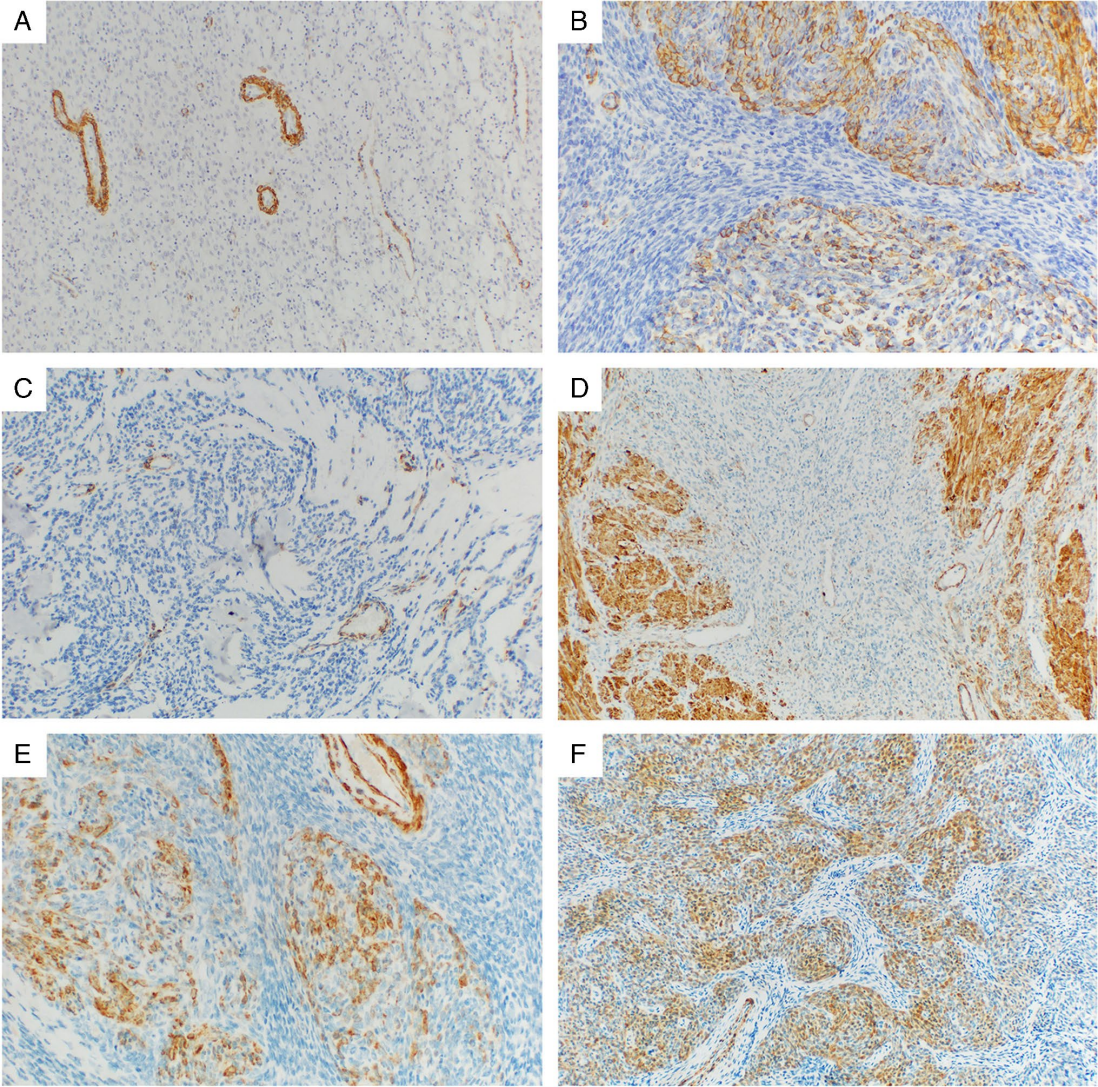
Examples of the expression of selected smooth muscle markers in different morphological variants of LG-ESS. **A** α-SMA in a usual LG-ESS, 100 × magnification (*JAZF1::SUZ12* fusion). **B** Focal irregular expression of α-SMA in LG-ESS with predominantly smooth-muscle morphology, 200 × magnification (*JAZF1::SUZ12* fusion). **C** Occasional rare expression of α-SMA in individual cells in a myxoid variant of LG-ESS (with positively staining vessels in the field), 100 × magnification (*JAZF1::SUZ12* fusion). **D** Almost complete negativity of transgelin in a usual LG-ESS (with positively staining admixed myometrium), 100 × magnification (no fusion detected). **E** Focal expression of transgelin smooth muscle variant of LG-ESS, 200 × magnification (*JAZF1::SUZ12* fusion, same case as 2B). **F** Transgelin in an LG-ESS with sex cord stromal-like morphology showing irregular but extensive weak to moderate expression, 100 × magnification (*JAZF1::SUZ12* fusion)

**Fig. 3:**
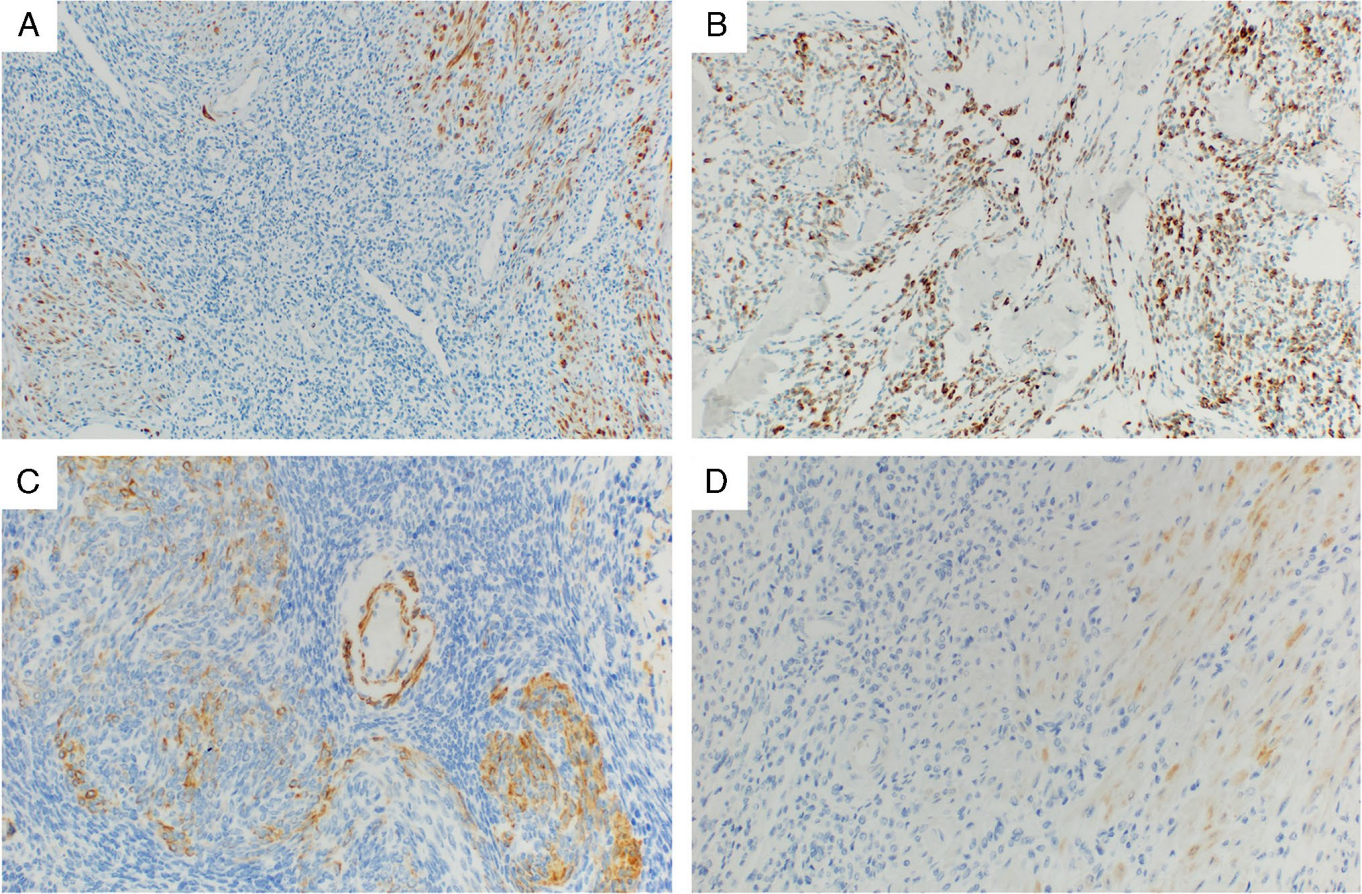
Examples of the expression of selected smooth muscle markers in different morphological variants of LG-ESS, continued. **A** Negativity of desmin in a usual variant of LG-ESS, 100 × magnification (no fusion detected, same case as 2D). **B** Disperse strong expression of desmin in a myxoid variant of LG-ESS, 100 × magnification (*JAZF1::SUZ12* fusion, same case as 2C). **C**) Focal weak to moderate expression of h-caldesmon in an LG-ESS with smooth muscle morphology, 200 × magnification (*JAZF1::SUZ12* fusion). **D** Smoothelin in a usual LG-ESS, 200 × magnification (no fusion detected, same case as 2D and 2G)

**Fig. 4:**
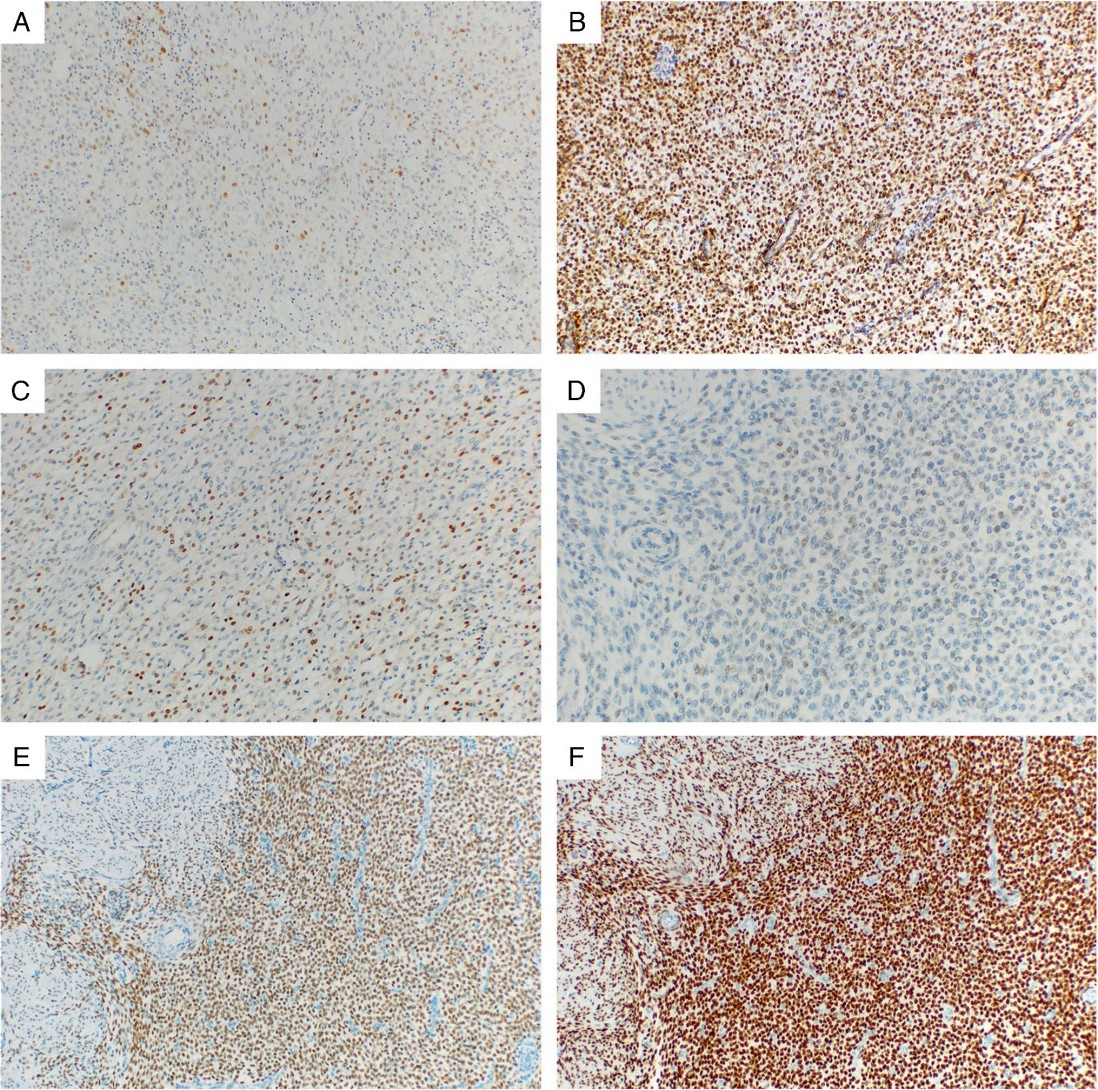
Examples of the expression of other selected IHC markers useful in the differential diagnosis of LG-ESS. **A** Extensive and nearly diffuse weak to moderate expression of Cyclin D1, 100 × magnification (*JAZF1::SUZ12* fusion). **B** Diffuse strong nuclear expression of WT1, 100 × magnification (*JAZF1::SUZ12* fusion). **C** Disperse expression of NTRK with variable (mostly moderate) intensity, 100 × magnification (*JAZF1::SUZ12* fusion). **D** The expression of p53 showing disperse, weak positivity of tumor cell—wild-type pattern of staining, 200 × magnification (*JAZF1::SUZ12* fusion). **E** Diffuse expression of ER of moderate intensity, 100 × magnification (*JAZF1::SUZ12* fusion). **F** Diffuse expression of PR, note the characteristic significantly stronger intensity of PR expression compared with ER (corresponding field from the same case as seen in 3E), 100 × magnification (*JAZF1::SUZ12* fusion)

A detailed overview of the IHC results is provided in Table 2. The IHC testing for all antibodies was not possible in all of the cases due to limited material. Representative images of selected IHC markers are provided in Figs. 1, 2, 3, and 4. The cohort consisted of 122 cases (83%) of LG-ESS with the usual morphological pattern, 5 cases (3%) with pure fibroblastic pattern, and a single case each (0.6%) displaying predominant smooth muscle-like morphology or a glandular pattern. Fourteen tumors showed a mixed pattern—most commonly usual + fibroblastic (*n* = 7.5%) and usual + smooth muscle-like (*n* = 2.1%), while 6 cases (4%) showed a sex cord-stromal component of variable extent. Two cases (1%) were made up of a mixture of fibroblastic and myxoid patterns, and one case displayed a combination of usual, fibroblastic, and myxoid morphology.

**Table 2:** Detailed overview of IHC expression of the antibodies most commonly used in the differential diagnosis of LG-ESS

IHC marker	Negative (0–1%) *n* (%)	Positive (≥ 1%) *n* (%)	Positivity 1 + (1–25%) *n* (%)	Positivity 2 + (26–50%) *n* (%)	Positivity 3 + (> 50%) *n* (%)	H-score mean (SD)	H-score median (range)
ER (*n* = 143)	25 (17%)	118 (83%)	6 (5%)	8 (7%)	104 (88%)	118 (87.3)	100 (0–300)
PR (*n* = 144)	11 (8%)	133 (92%)	8 (6%)	5 (4%)	120 (90%)	201 (106.5)	223 (0–300)
α-SMA (*n* = 142)	80 (56%)	62 (44%)	20 (32%)	7 (11%)	35 (56%)	46 (75.4)	0 (0–300)
Desmin (*n* = 141)	106 (75%)	35 (25%)	15 (43%)	6 (17%)	14 (40%)	24 (66.8)	0 (0–300)
H-caldesmon (*n* = 141)	121 (86%)	20 (14%)	12 (60%)	3 (15%)	5 (25%)	7 (28.0)	0 (0–200)
Calponin (*n* = 143)	94 (66%)	49 (34%)	22 (45%)	4 (8%)	23 (47%)	31 (72.7)	0 (0–300)
CD10 (*n* = 141)	20 (14%)	121 (86%)	6 (5%)	4 (3%)	111 (92%)	171 (110.9)	196 (0–300)
IFITM1 (*n* = 143)	45 (31%)	98 (69%)	28 (29%)	11 (11%)	59 (60%)	65 (77.2)	20 (0–300)
Transgelin (*n* = 141)	110 (78%)	31 (22%)	13 (42%)	6 (19%)	12 (39%)	16 (49.7)	0 (0–300)
BCOR (*n* = 142)	135 (95%)	7 (5%)	2 (29%)	1 (14%)	4 (57%)	3 (19.3)	0 (0–170)
BCORL1 (*n* = 141)	107 (76%)	34 (24%)	31 (91%)	2 (6%)	1 (3%)	2 (7.9)	0 (0–70)
NTRK nuclear (*n* = 145)	137 (94%)	8 (6%)	5 (63%)	3 (38%)	0 (0%)	2 (11.9)	0 (0–100)
NTRK cytopl. (*n* = 145)	141 (97%)	4 (3%)	2 (50%)	1 (25%)	1 (25%)	1 (8.8)	0 (0–100)
S-100 (*n* = 143)	143 (100%)	0 (0%)	0 (-)	0 (-)	0 (-)	0 (-)	0 (-)
HMB45 (*n* = 143)	140 (98%)	3 (2%)	3 (100%)	0 (0%)	0 (0%)	0 (1.8)	0 (0–20)
CD117 (*n* = 146)	145 (99%)	1 (1%)	1 (100%)	0 (0%)	0 (0%)	0 (1.7)	0 (0–20)
WT1 (*n* = 140)	84 (60%)	56 (40%)	18 (32%)	4 (7%)	34 (61%)	25 (43.3)	0 (0–200)
Smoothelin nuclear (*n* = 142)	141 (99%)	1 (1%)	1 (100%)	0 (0%)	0 (0%)	0 (0.1)	0 (0–1)
Smoothelin cytopl. (*n* = 142)	141 (99%)	1 (1%)	1 (100%)	0 (0%)	0 (0%)	0 (0.1)	0 (0–1)
Cyclin D1 (*n* = 141)	62 (44%)	79 (56%)	39 (49%)	26 (33%)	14 (18%)	25 (48.6)	2 (0–300)

*SD* standard deviation, *cytopl.* cytoplasmic. The number of analyzed cases for each stain differs due to the amount of available tissue. Percentages are rounded up/down. Percentages of 1 + /2 + /3 + positivity are counted only from the positive cases

As expected, the tumors showed high levels of expression of the endometrial stromal markers CD10 and IFITM1. CD10 expression was seen in 86% of cases, with a majority of the positive cases (92%) exhibiting diffuse, extensive staining of a high intensity (median H-score 196). The other marker of endometrial stromal differentiation IFITM1 was positive in a lower number of cases (69%) with a heterogenous extent of expression, which was mostly diffuse but of varying intensity. The expression of the standard smooth muscle markers (α-SMA, desmin, h-caldesmon, calponin, and transgelin) was present to a variable degree in all of the examined tumors, with α-SMA reaching the highest frequency of positive cases (44%). The extent of expression of these markers was typically on the opposite ends of the spectrum—either only rare and focal (with positively staining areas comprising less than 25% of the tumor tissue) or extensive and diffuse (with more than 50% of the tumor showing expression). However, the intensity of the smooth muscle marker expression was usually very low, with the highest average H-score of 46 (observed for α-SMA) and the median H-score 0 for all examined markers. Smoothelin showed expression in only two cases of LG-ESS, which was only focal and of a weak intensity. Hormone receptors were expressed in a high proportion of the tumors, with PR reaching a slightly higher frequency (92% vs. 83%) and also intensity of staining, compared with ER. Regarding the antibodies useful in differential diagnosis such as S-100, HMB45, and CD117, these showed almost complete negativity.

Of those markers not included in Table 2, all cases were p53 wild type, and ALK and ROS1 negative. The expression of SMARCB1 (INI1) and SMARCA4 (BRG1) was preserved, while seven cases showed an isolated loss of SMARCA2 (BRM). As the molecular profile of these tumors was known, none of them harbored *BRM* alterations.

The differences in expression of all the studied markers between the tumors with a confirmed fusion (*n* = 101) and those without a recurrent fusion (*n* = 32) were also analyzed (Supplementary Table 1). The results showed that only the endometrial stromal markers CD10 and IFITM1 differed significantly between the fusion positive and negative groups, with fusion positive tumors showing a more frequent positive expression of both CD10 (*p* < 0.001) and IFITM1 (*p* = 0.007).

Due to the imbalanced number of cases showing variant morphologies compared with the usual pattern, it was not possible to statistically analyze the differences in expression between these groups. Tumors with at least a component of fibroblastic morphology (*n* = 13) showed the expression of α-SMA, desmin, h-caldesmon, calponin, and transgelin in 5/13 (38%), 4/13 (31%), 3/13 (23%), 5/10 (50%), and 3/13 (23%) cases respectively. CD10 was positive in 11/13 (85%) cases and IFITM1 in 7/13 (54%) cases. Tumors with a sex cord-stromal component (*n* = 7) showed the expression of α-SMA, desmin, h-caldesmon, calponin, and transgelin in 5/7 (71%), 3/7 (43%), 2/7 (29%), 3/7 (43%), and 2/7 (29%) cases, respectively, while CD10 was positive in 6/7 (86%) cases and IFITM1 in 5/7 (71%) cases. The three tumors with smooth muscle morphology were all negative in desmin and h-caldesmon, and the expression of α-SMA, calponin, and transgelin was seen in 2/3 cases. All cases were CD10 positive, with IFITM1 expression in 2/3 cases.

## Discussion

Low grade endometrial stromal sarcoma presents a diagnostic challenge due to its significant morphological overlap with other spindle cell mesenchymal tumors of the uterus. Recent studies re-evaluating the pathological diagnoses of uterine mesenchymal tumors have reported that LG-ESS can be misdiagnosed in up to 20% of cases [22]. Current knowledge about the immunohistochemical profile of LG-ESS is derived from studies with a relatively low number of examined cases, with the largest immunohistochemically analyzed series including fewer than 50 cases [23]. Much of the published data comes from single-case studies and small antibody panels, limiting the availability of robust data. Additionally, LG-ESS is burdened with a relatively high interobserver variability, as some cases can only be reliably diagnosed using molecular testing. This introduces bias into older data, particularly due to evolving terminology and definitions, as some older studies only use the term “endometrial stromal sarcoma,” which makes the comparison of these results problematic. Given these limitations, our aim was to provide reliable data from a carefully selected, molecularly examined series of LG-ESS, the largest series described in the literature for IHC profiling to date. Reliable data on the immunoprofile of LG-ESS are especially critical in routine practice, particularly for cases with equivocal features or cases where only limited tumor tissue is available (e.g., small diagnostic biopsies or tru-cut biopsies).

The main IHC markers for diagnosing endometrial stromal tumors are CD10 and the more recently introduced marker IFITM1 [24, 25]. The expression of CD10 has long been established as one of the key diagnostic markers, as it is sensitive (75–100%) and has been reported in 258/294 (88%) of all cases of LG-ESS with the available IHC results to date. However, CD10 is not highly specific for LG-ESS, as a small portion of LG-ESS can be negative, especially in cases with poor fixation [26]. CD10 expression is also reported in up to 30% of smooth muscle tumors (including cellular leiomyoma), as well as in PEComa and the sarcomatous component of adenosarcoma [10, 27–30].

Currently, there is limited data on the expression of IFITM1 in LG-ESS. Two studies report IFITM1 expression in 83% (10/12) and 100% (16/16) of LG-ESS cases, compared with 30% (6/20) and 40% (12/30) of smooth muscle tumors [25, 31]. Both studies concluded that IFITM1 specificity (70% and 86,7%) surpasses CD10, although each study examined a relatively small series of cases. IFITM1 expression, while not entirely restricted to LG-ESS, tends to be weak and focal in smooth muscle tumors. Zhao et al. also emphasized IFITM1’s superior sensitivity and specificity over CD10, although also based on a small series [32]. Our study found IFITM1 expression in 98/143 cases (69%), mostly extensive or diffuse with variable intensity. IFITM1 seems to outperform CD10 in distinguishing LG-ESS from smooth muscle tumors; however, larger studies are still needed to confirm its utility. A useful approach would therefore include using both CD10 and IFITM1 as a part of the IHC panel. Interestingly, our study found that both CD10 and IFITM1 were significantly more commonly expressed in LG-ESS cases with a recurrent fusion, which underscores the diagnostic challenges of fusion-negative tumors given the lack of recurrent genetic alterations and potentially more equivocal IHC results.

Hormone receptors, specifically ER (ERα) and PR, are another crucial part of the diagnostic panel. The available literature indicates that ER is expressed in 84,5% of LG-ESS, with PR expression reaching 87% [15, 23, 33–38]. Most studies report more extensive, stronger PR expression than ER, which is consistent with our findings. The expression of androgen receptor (AR) has also been described in a high percentage of LG-ESS, though the number of studies is limited [35]. While ER and PR are typically less useful for distinguishing smooth muscle neoplasms, they can aid in ruling out other non-Müllerian origin tumors, particularly for extrauterine LG-ESS.

Hormone marker expression (especially ER and PR) may also have predictive significance, with their high expression rates in LG-ESS supporting hormone therapy such as high-dose progestins, aromatase inhibitors, or GnRH analogues as a viable treatment option [39–41]. A recent meta-analysis found that adjuvant hormone therapy can reduce recurrence risk in patients with FIGO stages I–II disease, but with no benefit concerning overall survival [42]. Another study suggested that hormone therapy can also reduce recurrence risk even in stages II–IV LG-ESS, recommending 12 months of high-dose progestin treatment post-surgery [43]. While the effectiveness of hormone therapy is debated, reporting tumor hormone status remains essential as it can help guide treatment decisions. Conflicting evidence also exists regarding ER/PR expression as a prognostic factor, requiring validation through a molecularly confirmed series [35, 43, 44].

In routine practice, one of the common diagnostic pitfalls lies in differentiating cellular leiomyoma (CL) from LG-ESS. The correct diagnosis of CL versus LG-ESS is of extreme clinical importance, given the different biological natures and behavior of these tumors. In which case, a combination of smooth muscle markers such as α-SMA, desmin, h-caldesmon, calponin, transgelin, and smoothelin, and endometrial stromal markers, such as CD10 and IFITM1, is essential. Smooth muscle markers are frequently positive in LG-ESS, especially in cases with smooth muscle differentiation [10, 12, 28, 30, 45, 46]. In contrast, endometrial stromal markers can be expressed in smooth muscle tumors, especially cellular leiomyomas. In our previous study on CL, the expression of CD10 was seen in 65% of cases, and the expression of IFITM1 in 36.5% of cases [13]. This can lead to a potential misclassification if an immunohistochemical (IHC) profile is not rigorously interpreted within the morphological context. The most expressed smooth muscle marker in LG-ESS is α-SMA (50% of cases reported in literature), closely followed by desmin (47%). While relatively common, the expression of all smooth muscle markers tends to be mostly focal and weak.

Based on the literature, h-caldesmon has emerged as the most specific marker for distinguishing smooth muscle tumors from LG-ESS [30, 46, 47]. The published data shows the expression of h-caldesmon in 9% of LG-ESS, and in our study, h-caldesmon was also the least expressed (14%) of the traditionally examined smooth muscle markers [10, 15, 27, 30, 32, 38, 46, 48–50]. Transgelin, an actin-binding protein of the calponin family, has similarly been noted as a highly sensitive and specific marker of smooth muscle differentiation. However, its expression in LG-ESS has been examined in just two studies, with a combined total of 32 cases [47, 51]. Our findings showed a 22% expression rate in LG-ESS, notably higher than previously published data, likely due to our larger series size. These findings highlight the need to validate the “characteristic” immunoprofile of LG-ESS to support the accurate diagnosis of challenging cases encountered in routine practice.

In our series, 14–44% of LG-ESS cases exhibited smooth muscle marker expression, underscoring the need for more specific antibodies. One such emerging marker could be smoothelin, initially identified in 1996 by van der Loop et al., which is a cytoskeletal protein primarily found in fully differentiated, contractile smooth muscle cells [52, 53]. This sets it apart from other smooth muscle markers such as calponin, α-SMA, smooth muscle myosin, and h-caldesmon, which are also present in less differentiated, proliferative smooth muscle cells. Additionally, smoothelin’s expression is notably lacking in cells that exhibit smooth muscle-like characteristics, such as myofibroblasts, myoepithelial cells, and striated muscle cells—cells that frequently express other smooth muscle proteins. Studies examining smoothelin’s utility in smooth muscle tumors of the gastrointestinal tract, uterus, and other soft tissues have indicated that cytoplasmic expression is highly sensitive and specific for benign leiomyomas [54, 55]. Notably, it has been suggested that the staining location (cytoplasmic vs. nuclear) may vary based on the biological behavior of smooth muscle tumors, as aberrant nuclear expression has been reported in a subset of leiomyosarcomas and occasionally in GIST. Our study is the first to examine smoothelin expression in LG-ESS, where we observed rare, moderate nuclear expression in one case and weak cytoplasmic expression in another. This suggests that smoothelin could be an extremely valuable marker in cases with equivocal results from the traditional smooth muscle markers. In our previous study, its expression was found in 61.5% of cellular leiomyomas, but it was commonly focal and weak, which limited its practical use [13].

A key takeaway from our findings is the awareness of smooth muscle marker expression in a subset of LG-ESS and the importance of interpreting results with caution, as they should always be evaluated together with endometrial stromal markers. When differentiating smooth muscle tumors from LG-ESS, a combined panel of high-sensitivity markers with specific markers (such as h-caldesmon, transgelin, and, potentially, smoothelin) is recommended, keeping in mind that α-SMA and desmin in particular do not serve as good discriminators between endometrial stromal and smooth muscle lineage. The staining’s extent and intensity are also essential; tumors with diffuse, strong expression of multiple smooth muscle markers should be, in a proper morphological context, classified as smooth muscle tumors, even if there is a focal expression of CD10 or IFITM1.

Of the other markers useful in differential diagnosis, cyclin D1 and BCOR are often part of the panel used for distinguishing LG-ESS from HG-ESS. Although HG-ESS typically exhibits a distinct morphology, rare cases of LG-ESS can present with epithelioid, round cell morphology which may lead to the consideration of HG-ESS. The reported rate of cyclin D1 expression in LG-ESS is generally low, reaching 28%. Our results indicate a relatively high proportion of LG-ESS with positive cyclin D1 expression (56%), though this expression was typically occasional or focal, and weak in intensity. Although a diffuse expression of cyclin D1 was seen in 14/141 (10%) cases, strong and diffuse cyclin D1 expression was present in only a single case (which showed a usual morphological pattern for LG-ESS). BCOR expression was significantly rarer (5%, 7/142) but, when present, the extent of staining ranged from occasional to nearly diffuse (albeit weak). Similarly, the expression of BCORL1, which can also be present in some cases of HG-ESS, was seen in 24% (34/141) of LG-ESS, where it was also only rare and weak. None of the cases with BCORL1 expression harbored a fusion involving the *BCORL1* gene. While BCOR and BCORL1 expression likely represent non-specific staining insufficient for HG-ESS diagnosis, they could pose a diagnostic challenge in certain ambiguous cases.

One of the less common differential diagnoses of LG-ESS is GIST, especially when found in extrauterine locations. In which case, a diagnostic panel of CD117 combined with CD10, ER, and PR is most effective, as CD34 can be positive in both LG-ESS and GIST [56]. Although some reports indicate a small portion of LG-ESS exhibit CD117 expression, the staining tends to be weak and focal [57, 58]. Consistent with prior reports, only a single case from our LG-ESS showed occasional, weak expression of CD117.

Another very important differential diagnosis is the entity of *KAT6B/A::KANSL1* fused sarcomas, described in 2022 by Agaimy et al. [16, 16, 17, 19]. According to the current knowledge, due to the overlapping features between endometrial stromal and smooth muscle differentiation, sarcomas with the *KAT6B/A::KANSL1* fusion cannot be diagnosed based only on the morphological and immunohistochemical features, and molecular testing is needed. The correct diagnosis is important in these cases as, despite their usually bland morphology, these tumors have propensity for aggressive behavior.

There are limitations to our study. The main one being the use of TMA, which brings the risk of underestimating/overestimating the IHC scoring. However, this risk was reduced by using two cores from each tumor to increase the amount of tissue, and this approach is widely used in the literature and allows for the examination of several markers on large sample collections. Additionally, a small portion of cases (*n* = 14) lack a known fusion status (as they could not undergo NGS RNA analysis due to insufficient amount or quality of the material), preventing molecular confirmation. Due to the low number of rare morphological variants, it was impossible to analyze the differences in expression between these and cases with conventional morphology.

## Conclusion

We have performed an extensive immunohistochemical analysis of a large series of molecularly examined 147 LG-ESS, using a wide panel of 24 antibodies. This study provides a more reliable immunohistochemical profile for LG-ESS, addressing the limitations of previous, smaller studies. Our findings, while not novel for most of the antibodies examined, provide an important context and validation of knowledge accumulated from studies which were limited and often on series which lack molecular confirmation. As such, our study is the largest to evaluate the immunoprofile of LG-ESS in general, with an emphasis on markers which have only recently come into diagnostic practice (such as transgelin and smoothelin). A crucial part of our results is the confirmation of the fact that due to the overlapping IHC profiles, it is impossible to reach a correct diagnosis using singular markers, and a panel of antibodies should always be used. We are aware that in some cases with overlapping morphological and immunohistochemical features, the correct diagnosis is only possible with molecular testing. However, 21% of LG-ESS in our series did not harbor a recurrent fusion—in these cases in particular the diagnosis will hinge on the assessment of the morphology and the immunohistochemical examination. Although there are still diagnostic challenges, this study provides critical data for improving diagnostic accuracy and guiding treatment. These findings validate existing knowledge, while contributing to a more standardized approach for diagnosing LG-ESS.

## Supplementary Information

Below is the link to the electronic supplementary material.

ESM 1 (DOCX 22.9 KB)

## Data Availability

The datasets used and/or analyzed during the current study are available from the corresponding author upon reasonable request.

## References

[1] WHO Classification of Tumours Editorial Board (2020) Female genital tumours. Lyon (France): International Agency for Research on Cancer. (WHO classification of tumours series, 5th ed.; vol. 4). https://publications.iarc.fr/592

[2] Amant F, Floquet A, Friedlander M, Kristensen G, Mahner S, Nam EJ, Powell MA, Ray-Coquard I, Siddiqui N, Sykes P, Westermann AM, Seddon B (2014) Gynecologic Cancer InterGroup (GCIG) consensus review for endometrial stromal sarcoma. Int J Gynecol Cancer 24:S67-72. 10.1097/IGC.0000000000000205 25033257 10.1097/IGC.0000000000000205

[3] Mayr D, Horn LC, Hiller GGR, Hohn AK, Schmoeckel E (2022) Endometrial and other rare uterine sarcomas : diagnostic aspects in the context of the 2020 WHO classification. Pathologe 43:183–195. 10.1007/s00292-022-01072-6 35362728 10.1007/s00292-022-01072-6

[4] Amador-Ortiz C, Roma AA, Huettner PC, Becker N, Pfeifer JD (2011) JAZF1 and JJAZ1 gene fusion in primary extrauterine endometrial stromal sarcoma. Hum Pathol 42:939–946. 10.1016/j.humpath.2010.11.001 21316079 10.1016/j.humpath.2010.11.001

[5] Zhu TH, Zhang FB, Yan H, Yu WY, Chen M, Guan YT (2022) A novel CDKN1A-JAZF1 gene fusion in low-grade endometrial stromal sarcoma arising from endometriosis in abdominal wall cesarean section scar: a case report and literature review. Taiwan J Obstet Gynecol 61:1082–1085. 10.1016/j.tjog.2022.04.010 36427980 10.1016/j.tjog.2022.04.010

[6] Dickson BC (2019) Beyond smooth muscle-other mesenchymal neoplasms of the uterus. Surg Pathol Clin 12:107–137. 10.1016/j.path.2018.10.005 30709439 10.1016/j.path.2018.10.005

[7] Akaev I, Yeoh CC, Rahimi S (2021) Update on endometrial stromal tumours of the uterus diagnostics (Basel) 11. 10.3390/diagnostics11030429 10.3390/diagnostics11030429 PMC8000701 33802452

[8] Conklin CM, Longacre TA (2014) Endometrial stromal tumors: the new WHO classification. Adv Anat Pathol 21:383–393. 10.1097/pap.0000000000000046 25299308 10.1097/PAP.0000000000000046

[9] Nucci MR (2016) Practical issues related to uterine pathology: endometrial stromal tumors. Mod Pathol 29(Suppl 1):S92-103. 10.1038/modpathol.2015.140 26715176 10.1038/modpathol.2015.140

[10] Oliva E, Young RH, Amin MB, Clement PB (2002) An immunohistochemical analysis of endometrial stromal and smooth muscle tumors of the uterus: a study of 54 cases emphasizing the importance of using a panel because of overlap in immunoreactivity for individual antibodies. Am J Surg Pathol 26:403–412. 10.1097/00000478-200204000-00001 11914617 10.1097/00000478-200204000-00001

[11] Richmond AM, Rohrer AJ, Davidson SA, Post MD (2017) Low-grade endometrial stromal sarcoma with extensive sex cord differentiation, heterologous elements, and complex atypical hyperplasia: Case report and review of literature Gynecol. Oncol Rep 19:34–38. 10.1016/j.gore.2016.12.002 10.1016/j.gore.2016.12.002 PMC5199152 28054022

[12] Agoff SN, Grieco VS, Garcia R, Gown AM (2001) Immunohistochemical distinction of endometrial stromal sarcoma and cellular leiomyoma. Appl Immunohistochem Mol Morphol 9:164–169. 10.1097/00129039-200106000-00009 11396634 10.1097/00129039-200106000-00009

[13] Dundr P, Gregova M, Hojny J, Krkavcova E, Michalkova R, Nemejcova K, Bartu M, Hajkova N, Laco J, Mara M, Richtarova A, Zima T, Struzinska I (2022) Uterine cellular leiomyomas are characterized by common HMGA2 aberrations, followed by chromosome 1p deletion and MED12 mutation: morphological, molecular, and immunohistochemical study of 52 cases. Virchows Arch 480:281–291. 10.1007/s00428-021-03217-z 34626221 10.1007/s00428-021-03217-z

[14] Flidrova M, Hajkova N, Hojny J, Dvorak J, Michalkova R, Krkavcova E, Laco J, McCluggage WG, Giordano G, Silini EM, Michalova K, Bizon M, Nemejcova K, Dundr P, Kendall Bartu M (2024) Unraveling the molecular landscape of uterine tumor resembling ovarian sex cord tumor: insights from a clinicopathological, morphologic, immunohistochemical, and molecular analysis of 35 cases. Mod Pathol 37:100611. 10.1016/j.modpat.2024.100611 39265954 10.1016/j.modpat.2024.100611

[15] Hwang H, Matsuo K, Duncan K, Pakzamir E, Pham HQ, Correa A, Fedenko A, Mhawech-Fauceglia P (2015) Immunohistochemical panel to differentiate endometrial stromal sarcoma, uterine leiomyosarcoma and leiomyoma: something old and something new. J Clin Pathol 68:710–717. 10.1136/jclinpath-2015-202915 25991737 10.1136/jclinpath-2015-202915 PMC7528441

[16] Agaimy A, Clarke BA, Kolin DL, Lee CH, Lee JC, McCluggage WG, Poschke P, Stoehr R, Swanson D, Turashvili G, Beckmann MW, Hartmann A, Antonescu CR, Dickson BC (2022) Recurrent KAT6B/A::KANSL1 fusions characterize a potentially aggressive uterine sarcoma morphologically overlapping with low-grade endometrial stromal sarcoma. Am J Surg Pathol 46:1298–1308. 10.1097/PAS.0000000000001915 35575789 10.1097/PAS.0000000000001915 PMC9388494

[17] Trecourt A, Azmani R, Hostein I, Blanchard L, Le Loarer F, Bourdon A, Alame M, Nadaud B, Mayer L, Rebier F, Larmonier C, Moura MS, Soubeyran I, Hartog C, Ray-Coquard I, Treilleux I, Devouassoux-Shisheboran M, Croce S (2023) The KAT6B::KANSL1 fusion defines a new uterine sarcoma with hybrid endometrial stromal tumor and smooth muscle tumor features. Mod Pathol 36:100243. 10.1016/j.modpat.2023.100243 37307879 10.1016/j.modpat.2023.100243

[18] Kommoss FKF, Charbel A, Kolin DL, Howitt BE, Kobel M, Lee JC, McCluggage WG, Agaimy A, Dickson BC, von Deimling A, Lee CH (2024) Uterine mesenchymal tumours harboring the KAT6B/A::KANSL1 gene fusion represent a distinct type of uterine sarcoma based on DNA methylation profiles. Virchows Arch 485:793–803. 10.1007/s00428-024-03935-0 39392508 10.1007/s00428-024-03935-0 PMC11564218

[19] Dundr P, Dvorak J, Krausova M, Hojny J, Hajkova N, Struzinska I, Nemejcova K, Ondic O, Michal M, Michalova K, Berjon A, Jedryka M, Ksiazek M, Poprawski T, Rys J, Volodko N, Zapardiel I, Zima T, Cibula D, Poncova R, Matej R, Kendall Bartu M (2024) Uterine sarcoma with KAT6B/A::KANSL1 fusion: a molecular and clinicopathological study on 9 cases. Virchows Arch. 10.1007/s00428-024-03994-3 10.1007/s00428-024-03994-3 PMC11950137 39627614

[20] Dundr P, Matej R, Hojny J, Hajkova N, Nemejcova K, Kendall Bartu M (2024) The spectrum of fusions occurring in non-smooth muscle mesenchymal uterine tumors: a review of the current knowledge. Arch Pathol Lab Med. 10.5858/arpa.2023-0324-RA 10.5858/arpa.2023-0324-RA 38484759

[21] Hirsch FR, Varella-Garcia M, Bunn PA Jr, Di Maria MV, Veve R, Bremmes RM, Baron AE, Zeng C, Franklin WA (2003) Epidermal growth factor receptor in non-small-cell lung carcinomas: correlation between gene copy number and protein expression and impact on prognosis. J Clin Oncol 21:3798–3807. 10.1200/JCO.2003.11.069 12953099 10.1200/JCO.2003.11.069

[22] Yoshida H, Kikuchi A, Tsuda H, Sakamoto A, Fukunaga M, Kaku T, Yoshida M, Shikama A, Kogata Y, Terao Y, Tanikawa M, Yasuoka T, Chiyoda T, Miyamoto T, Okadome M, Nakamura T, Enomoto T, Konno Y, Yahata H, Hirata Y, Aoki Y, Tokunaga H, Usui H, Yaegashi N (2022) Discrepancies in pathological diagnosis of endometrial stromal sarcoma: a multi-institutional retrospective study from the Japanese clinical oncology group. Hum Pathol 124:24–35. 10.1016/j.humpath.2022.03.007 35339567 10.1016/j.humpath.2022.03.007

[23] Kolin DL, Nucci MR, Turashvili G, Song SJ, Corbett-Burns S, Cesari M, Chang MC, Clarke B, Demicco E, Dube V, Lee CH, Rouzbahman M, Shaw P, Cin PD, Swanson D, Dickson BC (2024) Targeted RNA sequencing highlights a diverse genomic and morphologic landscape in low-grade endometrial stromal sarcoma, including novel fusion genes. Am J Surg Pathol 48:36–45. 10.1097/PAS.0000000000002142 37867306 10.1097/PAS.0000000000002142

[24] Park HJ, Kuk IS, Kim JH, Kim JH, Song SJ, Choi BC, Kim B, Kim NH, Song H (2011) Characterisation of mouse interferon-induced transmembrane protein-1 gene expression in the mouse uterus during the oestrous cycle and pregnancy. Reprod Fertil Dev 23:798–808. 10.1071/RD10086 21791181 10.1071/RD10086

[25] Parra-Herran CE, Yuan L, Nucci MR, Quade BJ (2014) Targeted development of specific biomarkers of endometrial stromal cell differentiation using bioinformatics: the IFITM1 model. Mod Pathol 27:569–579. 10.1038/modpathol.2013.123 24072182 10.1038/modpathol.2013.123

[26] Tang Y, Chen Y, Tian L, Chen J, Yang P, Zhang D, Cui Q, Zhao L, Li L (2020) Vaginal low-grade endometrial stromal sarcoma: an extremely rare case report and review of the literature. Int J Gynecol Pathol 39:447–451. 10.1097/PGP.0000000000000626 31569185 10.1097/PGP.0000000000000626

[27] Abeler VM, Nenodovic M (2011) Diagnostic immunohistochemistry in uterine sarcomas: a study of 397 cases. Int J Gynecol Pathol 30:236–243. 10.1097/PGP.0b013e318200caff 21464730 10.1097/PGP.0b013e318200caff

[28] Baker P, Oliva E (2007) Endometrial stromal tumours of the uterus: a practical approach using conventional morphology and ancillary techniques. J Clin Pathol 60:235–243. 10.1136/jcp.2005.031203 17347285 10.1136/jcp.2005.031203 PMC1860562

[29] Mittal K, Soslow R, McCluggage WG (2008) Application of immunohistochemistry to gynecologic pathology. Arch Pathol Lab Med 132:402–423. 10.5858/2008-132-402-AOITGP 18318583 10.5858/2008-132-402-AOITGP

[30] Rush DS, Tan J, Baergen RN, Soslow RA (2001) h-Caldesmon, a novel smooth muscle-specific antibody, distinguishes between cellular leiomyoma and endometrial stromal sarcoma. Am J Surg Pathol 25:253–258. 10.1097/00000478-200102000-00014 11176075 10.1097/00000478-200102000-00014

[31] Busca A, Gulavita P, Parra-Herran C, Islam S (2017) IFITM1 outperforms CD10 in differentiating low-grade endometrial stromal sarcomas from smooth muscle neoplasms of the uterus. Int J Gynecol Pathol. 10.1097/PGP.0000000000000424 10.1097/PGP.0000000000000424 28700435

[32] Zhao W, Cui M, Zhang R, Shen X, Xiong X, Ji X, Tao L, Jia W, Pang L, Sun Z, Wang C, Zou H (2021) IFITM1, CD10, SMA, and h-caldesmon as a helpful combination in differential diagnosis between endometrial stromal tumor and cellular leiomyoma. BMC Cancer 21:1047. 10.1186/s12885-021-08781-w 34556086 10.1186/s12885-021-08781-w PMC8461929

[33] Jakate K, Azimi F, Ali RH, Lee CH, Clarke BA, Rasty G, Shaw PA, Melnyk N, Huntsman DG, Laframboise S, Rouzbahman M (2013) Endometrial sarcomas: an immunohistochemical and JAZF1 re-arrangement study in low-grade and undifferentiated tumors. Mod Pathol 26:95–105. 10.1038/modpathol.2012.136 22918161 10.1038/modpathol.2012.136

[34] Kurihara S, Oda Y, Ohishi Y, Iwasa A, Takahira T, Kaneki E, Kobayashi H, Wake N, Tsuneyoshi M (2008) Endometrial stromal sarcomas and related high-grade sarcomas: immunohistochemical and molecular genetic study of 31 cases. Am J Surg Pathol 32:1228–1238. 10.1097/PAS.0b013e31816a3b42 18580489 10.1097/PAS.0b013e31816a3b42

[35] Park JY, Baek MH, Park Y, Kim YT, Nam JH (2018) Investigation of hormone receptor expression and its prognostic value in endometrial stromal sarcoma. Virchows Arch 473:61–69. 10.1007/s00428-018-2358-5 29869299 10.1007/s00428-018-2358-5

[36] Rahimi S, Akaev I, Marani C, Chopra M, Yeoh CC (2019) Immunohistochemical expression of different subtypes of cytokeratins by endometrial stromal sarcoma. Appl Immunohistochem Mol Morphol 27:466–470. 10.1097/PAI.0000000000000642 29406332 10.1097/PAI.0000000000000642

[37] Vera AA, Guadarrama MB (2011) Endometrial stromal sarcoma: clinicopathological and immunophenotype study of 18 cases. Ann Diagn Pathol 15:312–317. 10.1016/j.anndiagpath.2011.01.008 21652246 10.1016/j.anndiagpath.2011.01.008

[38] Zhu XQ, Shi YF, Cheng XD, Zhao CL, Wu YZ (2004) Immunohistochemical markers in differential diagnosis of endometrial stromal sarcoma and cellular leiomyoma. Gynecol Oncol 92:71–79. 10.1016/j.ygyno.2003.08.038 14751141 10.1016/j.ygyno.2003.08.038

[39] Leunen M, Breugelmans M, De Sutter P, Bourgain C, Amy JJ (2004) Low-grade endometrial stromal sarcoma treated with the aromatase inhibitor letrozole. Gynecol Oncol 95:769–771. 10.1016/j.ygyno.2004.07.063 15582003 10.1016/j.ygyno.2004.07.063

[40] Maenohara S, Fujimoto T, Okadome M, Sonoda K, Taguchi K, Saito T (2020) Potential alternative progestin therapy for low-grade endometrial stromal sarcoma: a case report Gynecol. Oncol Rep 34:100634. 10.1016/j.gore.2020.100634 10.1016/j.gore.2020.100634 PMC7486439 32953963

[41] Thiel FC, Halmen S (2018) Low-grade endometrial stromal sarcoma-a review. Oncol Res Treat 41:687–692. 10.1159/000494225 30317238 10.1159/000494225

[42] Cui R, Cao G, Bai H, Zhang Z (2019) The clinical benefits of hormonal treatment for LG-ESS: a meta-analysis. Arch Gynecol Obstet 300:1167–1175. 10.1007/s00404-019-05308-4 31583462 10.1007/s00404-019-05308-4

[43] Huang X, Peng P (2022) Hormone therapy reduces recurrence in stage ii-iv uterine low-grade endometrial stromal sarcomas: a retrospective cohort study. Front Oncol 12:922757. 10.3389/fonc.2022.922757 35837098 10.3389/fonc.2022.922757 PMC9275776

[44] Cade TJ, Quinn MA, Rome RM, Polyakov A (2014) Prognostic significance of steroid receptor positivity and adjuvant progestogen use in endometrial stromal sarcoma. Aust N Z J Obstet Gynaecol 54:453–456. 10.1111/ajo.12245 25287561 10.1111/ajo.12245

[45] Chu PG, Arber DA, Weiss LM, Chang KL (2001) Utility of CD10 in distinguishing between endometrial stromal sarcoma and uterine smooth muscle tumors: an immunohistochemical comparison of 34 cases. Mod Pathol 14:465–471. 10.1038/modpathol.3880335 11353058 10.1038/modpathol.3880335

[46] Nucci MR, O’Connell JT, Huettner PC, Cviko A, Sun D, Quade BJ (2001) h-Caldesmon expression effectively distinguishes endometrial stromal tumors from uterine smooth muscle tumors. Am J Surg Pathol 25:455–463. 10.1097/00000478-200104000-00004 11257619 10.1097/00000478-200104000-00004

[47] Robin YM, Penel N, Perot G, Neuville A, Velasco V, Ranchere-Vince D, Terrier P, Coindre JM (2013) Transgelin is a novel marker of smooth muscle differentiation that improves diagnostic accuracy of leiomyosarcomas: a comparative immunohistochemical reappraisal of myogenic markers in 900 soft tissue tumors. Mod Pathol 26:502–510. 10.1038/modpathol.2012.192 23174934 10.1038/modpathol.2012.192

[48] D’Angelo E, Spagnoli LG, Prat J (2009) Comparative clinicopathologic and immunohistochemical analysis of uterine sarcomas diagnosed using the World Health Organization classification system. Hum Pathol 40:1571–1585. 10.1016/j.humpath.2009.03.018 19540555 10.1016/j.humpath.2009.03.018

[49] Kim GW, Baek SK, Han JJ, Kim HJ, Sung JY, Maeng CH (2022) Pulmonary metastasizing low-grade endometrial stromal sarcoma: case report and review of diagnostic pitfalls diagnostics (Basel) 12. 10.3390/diagnostics12020271 10.3390/diagnostics12020271 PMC8871004 35204363

[50] Yano Y, Yamasaki Y, Yamanaka K, Nishimoto M, Nagamata S, Terai Y (2023) A case of a recurrent low-grade endometrial stromal sarcoma extending to the inferior vena cava (IVC) after the primary fertility-sparing surgery. Int J Surg Case Rep 111. 10.1016/j.ijscr.2023.108857 37741074 10.1016/j.ijscr.2023.108857 PMC10520521

[51] Tawfik O, Rao D, Nothnick WB, Graham A, Mau B, Fan F (2014) Transgelin, a novel marker of smooth muscle differentiation, effectively distinguishes endometrial stromal tumors from uterine smooth muscle tumors. Int J Gynecol Obstet Reprod Med Res 1:26–31 26023684 PMC4443873

[52] Kramer J, Aguirre-Arteta AM, Thiel C, Gross CM, Dietz R, Cardoso MC, Leonhardt H (1999) A novel isoform of the smooth muscle cell differentiation marker smoothelin. J Mol Med (Berl) 77:294–298. 10.1007/s001090050352 10023782 10.1007/s001090050352

[53] van der Loop FT, Schaart G, Timmer ED, Ramaekers FC, van Eys GJ (1996) Smoothelin, a novel cytoskeletal protein specific for smooth muscle cells. J Cell Biol 134:401–411. 10.1083/jcb.134.2.401 8707825 10.1083/jcb.134.2.401 PMC2120883

[54] Coco DP, Hirsch MS, Hornick JL (2009) Smoothelin is a specific marker for smooth muscle neoplasms of the gastrointestinal tract. Am J Surg Pathol 33:1795–1801. 10.1097/pas.0b013e3181b76477 19950405 10.1097/pas.0b013e3181b76477

[55] Mayhall KG Jr, Oertling E, Lewin E, Schmieg J, LeBeau H, Wu T, Crawford B (2019) The use of smoothelin and other antibodies in the diagnosis of uterine and soft tissue smooth muscle tumors. Appl Immunohistochem Mol Morphol 27:386–391. 10.1097/PAI.0000000000000619 29189258 10.1097/PAI.0000000000000619

[56] Ahn SR, Lee JH (2020) Low-grade endometrial stromal sarcoma presenting as a sigmoid mass. Korean J Gastroenterol 76:322–326. 10.4166/kjg.2020.114 33361707 10.4166/kjg.2020.114 PMC12286495

[57] Klein WM, Kurman RJ (2003) Lack of expression of c-kit protein (CD117) in mesenchymal tumors of the uterus and ovary. Int J Gynecol Pathol 22:181–184. 10.1097/00004347-200304000-00011 12649674 10.1097/00004347-200304000-00011

[58] Nakayama M, Mitsuhashi T, Shimizu Y, Ban S, Ogawa F, Ishihara O, Shimizu M (2006) Immunohistochemical evaluation of KIT expression in sarcomas of the gynecologic region. Int J Gynecol Pathol 25:70–76. 10.1097/01.pgp.0000183047.45459.36 16306788 10.1097/01.pgp.0000183047.45459.36

