# Supplementary material for: Immunohistochemical analysis of 147 cases of low-grade endometrial stromal sarcoma: refining the immunohistochemical profile of LG-ESS on a large, molecularly confirmed series
Source: Virchows Arch. 2025 Jan 21;486(6):1289–304. doi: 10.1007/s00428-025-04026-4 (PMC12213977; doi:10.1007/s00428-025-04026-4)
Supplement: Supplementary file 1 — (DOCX 22.9 KB) [file 428_2025_4026_MOESM1_ESM.docx]

**Supplementary Table 1**: The expression of selected IHC markers in relation to the fusion status of LG-ESS with known RNA NGS profile.

| **IHC marker** | **Fusion negative  n (%)** | **Fusion positive n (%)** | ***p*-value** |
| --- | --- | --- | --- |
| **ER** |  |  | 0.054^p^ |
| Negative (<5%) | 10 (38%) | 16 (62%) |  |
| Positive (≥5%) | 21 (20%) | 82 (80%) |  |
| **PR** |  |  | > 0.995^f^ |
| Negative (<5%) | 3 (23%) | 10 (77%) |  |
| Positive (≥5%) | 28 (24%) | 90 (76%) |  |
| **α-SMA** |  |  | 0.663 ^p^ |
| Negative (<5%) | 16 (23%) | 55 (77%) |  |
| Positive (≥5%) | 15 (26%) | 43 (74%) |  |
| **Desmin** |  |  | 0.064 ^p^ |
| Negative (<5%) | 20 (20%) | 80 (80%) |  |
| Positive (≥5%) | 10 (37%) | 17 (63%) |  |
| **H-caldesmon** |  |  | 0.376 ^f^ |
| Negative (<5%) | 25 (23%) | 85 (77%) |  |
| Positive (≥5%) | 6 (33%) | 12 (67%) |  |
| **Calponin** |  |  | 0.061 ^p^ |
| Negative (<5%) | 18 (20%) | 74 (80%) |  |
| Positive (≥5%) | 13 (35%) | 24 (65%) |  |
| **CD10** |  |  | **< 0.001** ^f^ |
| Negative (<5%) | 14 (67%) | 7 (33%) |  |
| Positive (≥5%) | 16 (15%) | 90 (85%) |  |
| **IFITM1** |  |  | **0.007** ^p^ |
| Negative (<5%) | 17 (38%) | 28 (62%) |  |
| Positive (≥5%) | 14 (16%) | 71 (84%) |  |
| **Transgelin** |  |  | 0.654 ^p^ |
| Negative (<5%) | 23 (23%) | 78 (77%) |  |
| Positive (≥5%) | 7 (27%) | 19 (73%) |  |
| **BCOR** |  |  | 0.592 ^f^ |
| Negative (<5%) | 29 (23%) | 96 (77%) |  |
| Positive (≥5%) | 2 (40%) | 3 (60%) |  |
| **BCORL1** |  |  | > 0.995 ^f^ |
| Negative (<5%) | 28 (24%) | 90 (76%) |  |
| Positive (≥5%) | 2 (22%) | 7 (78%) |  |
| **NTRK nuclear** |  |  | > 0.995 ^f^ |
| Negative (<5%) | 31 (25%) | 94 (75%) |  |
| Positive (≥5%) | 1 (17%) | 5 (83%) |  |
| **NTRK cytopl.** |  |  | > 0.995 ^f^ |
| Negative (<5%) | 31 (24%) | 97 (76%) |  |
| Positive (≥5%) | 1 (33%) | 2 (67%) |  |
| **S-100** |  |  | NULL |
| Negative (<5%) | 30 (23%) | 99 (77%) |  |
| Positive (≥5%) | 0 (0%) | 0 (0%) |  |
| **HMB45** |  |  | > 0.995 ^f^ |
| Negative (<5%) | 30 (24%) | 97 (76%) |  |
| Positive (≥5%) | 0 (0%) | 2 (100%) |  |
| **CD117** |  |  | 0.235 ^f^ |
| Negative (<5%) | 30 (23%) | 101 (77%) |  |
| Positive (≥5%) | 1 (100%) | 0 (0%) |  |
| **WT1** |  |  | 0.237 ^p^ |
| Negative (<5%) | 22 (27%) | 59 (73%) |  |
| Positive (≥5%) | 8 (18%) | 37 (82%) |  |
| **Smoothelin nuclear** |  |  | NULL |
| Negative (<5%) | 31 (24%) | 97 (76%) |  |
| Positive (≥5%) | 0 (0%) | 0 (0%) |  |
| **Smoothelin cyto**pl. |  |  | > 0.995 ^f^ |
| Negative (<5%) | 31 (24%) | 96 (76%) |  |
| Positive (≥5%) | 0 (0%) | 1 (100%) |  |
| **Cyclin D1** |  |  | > 0.995 ^f^ |
| Negative (<5%) | 13 (18%) | 60 (82%) |  |
| Positive (≥5%) | 17 (32%) | 36 (68%) |  |

cytopl. – cytoplasmic, *p-*values are based on Pearson’s chi-squared test (^p^) or Fisher´s Exact test (^f^) based on expected values.
